# A Pilot Study of Using Smartphone Application vs. Routine Follow-Up for Patient Care in Advanced Non-Small Cell Lung Cancer During the COVID-19 Pandemic Era

**DOI:** 10.3389/fmedt.2022.900172

**Published:** 2022-06-21

**Authors:** Naiyarat Prasongsook, Kasan Seetalarom, Siriwimon Saichaemchan, Kittipong Udomdamrongkul

**Affiliations:** Division of Medical Oncology, Department of Medicine, Phramongkutklao Hospital, Bangkok, Thailand

**Keywords:** advanced NSCLC, patients-reported outcomes, COVID-19 pandemic, mobile application, cancer care, survival outcome

## Abstract

**Introduction:**

Cancer care monitoring should be adapted regarding COVID-19 pandemic preparedness plans. Lung Cancer Care application was a mobile application program to monitor adverse events and report outcomes. This study is aimed to invent a new mobile application evaluating patient-reported outcome (PRO) for patients with non-small cell lung cancer (NSCLC) and to evaluate the validity of a mobile application, particularly during the COVID-19 pandemic era.

**Methods:**

The validity of the application was tested, and Functional Assessment of Cancer Therapy-Lung (FACT-L) questionnaires were contained in the mobile application-based PRO. Patients were randomly assigned to use mobile application-based PRO vs. routine follow-up. The primary end point was to compare the quality of life (QoL) scores between two groups. A secondary end point was overall survival (OS) and the outcomes of progressive disease (PD) between the two groups.

**Results:**

In total, 33 patients with advanced NSCLC were enrolled. Patients in the mobile application group had higher FACT-L scores at 3 months than patients with a routine follow-up arm (106 ± 5.97 vs. 99.96 ± 5.74, *p*-value = 0.07). The median follow-up time was 5.43 months; patients with mobile application had an insignificant increase in median OS when compared with patients using routine follow-up (4.53 vs. 2.93 months, *p*-value = 0.85). The sensitivity, specificity, positive predictive value (PPV), and negative predictive (NPV) value of this application for predicting disease progression were 50, 83.3, 66.7, and 70%, respectively.

**Conclusion:**

Self-reported symptoms by Lung Cancer Care application improved QoL and were similar in monitoring outcomes to face-to-face follow-up. This tool is applicable for patients with cancer to make monitoring as safe as possible for physical distancing during the COVID-19 pandemic era.

## Introduction

The rapidly spreading coronavirus disease 2019 (COVID-19) acute respiratory pandemic has impacted patients with cancer. One publication from China reported that COVID-19 patients with cancer had 3.5 times higher risk of mortality than patients without cancer (1). In particular, the first results from the Thoracic cancERs international COVID-19 coLLaboraTion (TERAVOLT) registry revealed an unexpectedly high mortality rate of COVID-19 among patients with thoracic cancers, for which 75.5% of those with thoracic malignancy involved advanced stage non-small cell lung cancer (NSCLC) (2). Therefore, the adaptive routine practice may be needed to deal with the challenges of cancer care.

Recently, advanced technologies in computers and smartphones have become widely used, particularly among clinicians for monitoring and managing side effects from systemic treatments. For example, Denis et al. (3, 4) reported that using a weekly electronic-based self-evaluation of six symptoms (such as weight loss, fatigue, loss of appetite, pain, cough, and breathlessness) was feasible and accurate for earlier detection of lung cancer relapse or progression. Moreover, some studies have demonstrated combining this tool and routine follow-up protocol improved quality of life (QoL), progression-free survival, and overall survival (OS) when compared to patients with lung cancer who had merely routine follow-up protocol (5, 6). Therefore, patients' self-reported symptoms have recently become interesting in the oncology field for potential improvement in the efficacy of patient care (7–9).

We developed a novel mobile application, namely, Lung Cancer Care for self-reporting self-assessment for QoL and side effects from systemic treatment, particularly among patients receiving systemic treatments, such as chemotherapy, targeted therapies, and immunotherapy. We tested the hypothesis that the Lung Cancer Care application improves QoL and survival among patients with advanced NSCLC when compared with patients under routine care. Importantly, we hope this tool will minimize face-to-face contact and reduce spending unnecessary time in a hospital to reduce the risk of COVID-19 exposure.

## Methods

### Study Populations

Patients with histologically confirmed NSCLC receiving first- or second-line systemic treatment were enrolled. Additional inclusion criteria included age at least 18 years old, Eastern Cooperative Oncology Group (ECOG) performance status (PS) ≤2, white blood cell count ≥3,000 cell/mm^3^, hemoglobin ≥10 g/dl, platelet count ≥100,000 cells/mm^3^, serum creatinine ≤1.5 mg/dl, total bilirubin ≤2 mg/dl, and aspartate aminotransferase (AST) ≤3 times the upper limit of normal (ULN) for those patients without liver metastasis or ≤5 times the ULN for those patients with liver metastasis. Exclusion criteria comprised patients with advanced NSCLC who received more than 2 prior regimens of chemotherapy, targeted therapy or immunotherapy, had second primary cancer, or both clinical or imaging confirmed brain metastasis. In addition, patients with an active infection or uncontrolled medical conditions, e.g., unstable angina, uncontrolled hypertension, history of congestive heart failure, or history of myocardial infarction, were excluded.

### Study Design

#### In Cohort 1 (Tool Construction and Validation)

The prefinal version of the translated questionnaire was pilot tested on the intended respondents. After completing the translated questionnaire, the respondent was asked verbally by an interviewer to elaborate what they thought concerning each questionnaire item and what their corresponding response meant. This process was repeated a few times to finalize the final version of the smartphone application (additional details are provided in the Supplementary Material regarding the electronic infrastructure).

#### In Cohort 2

Patients in this cohort were enrolled and analyzed separately from patients in cohort 1.

The study design in this cohort was a randomized, double blind, and placebo-controlled trial conducted among patients with advanced NSCLC who received specific treatment in Phramongkutklao Hospital. The study was approved by the Phramongkutklao College of Medicine Ethics Committee and conducted according to the International Conference on Harmonization (ICH) on Good Clinical Practice (GCP) requirements. All patients were provided and signed their informed consent forms.

Four-by-four block randomization was used to divide eligible patients into two arms, who were randomized in 1:1 ratio by computer program into patients using a mobile application and patients monitored using routine care. Random assignment was performed on day 1 of the first cycle (day) for the first or second line of systemic treatment. All patients signed written informed consent forms. The study followed the guidelines of our institutional ethical committee.

- Group 1: The intervention arm was defined as using the Lung Cancer Care application. The Functional Assessment of Cancer Therapy-Lung (FACT-L) questionnaires from the mobile application were completed by patients at baseline (before treatment), the first day of the first follow-up (weeks 3–4), and the third follow-up (3 months after randomization) of the treatment.
- Group 2: Patients with routine follow-up care were assessed their QoL from paper-based validation of the Thai version of FACT-L questionnaires at baseline (before treatment), the first day of the first follow-up (weeks 3–4), and third follow-up (3 months after randomization) of the treatment by the same physician on the group 1.
- All patients in both groups were evaluated by a standard protocol for imaging assessment (chest CT scan every 8–12 weeks or as clinically indicated).

### Outcome Measures

#### Cohort 1: Tool Construction and Validation

The primary study end point was QoL, which was assessed using the FACT-L containing the Lung Cancer Care application. The five categorized domains included physical wellbeing (PWB), social wellbeing (SWB), emotional wellbeing (EWB), functional wellbeing (FWB), and lung cancer subscale (LCS). The questionnaire is well validated and has been used widely in lung cancer trials. The validated Thai version of the FACT-L with corresponding psychometric properties of the original was used and has been the main part of the mobile application in this study.

The content for assessing the Lung Cancer Care application consisted of two parts, which were QoL assessment using the validation of the Thai version of FACT-L and symptoms related to adverse events from systemic treatment using the Common Terminology Criteria for Adverse Events (CTCAE) version 4.0 (Table 1). However, we used only FACT-L data from this application to validate the tool and analyze the outcomes. This application was free to download on the android system *via* a play store using the keywords “Lung Cancer Care” for searching.

**Table 1:** Five symptoms evaluated in the application scoring from 0 to 3 for each question.

**Symptom**	**Score for symptoms**			
	**No**	**Low**	**Medium**	**High**
Fatigue	0	1	2	3
Appetite	0	1	2	3
Cough	0	1	2	3
Breathlessness	0	1	2	3
Pain	0	1	2	3

For translating the tool, the FACT-L questionnaire was initially translated from the original language to Thai (forward translation). Moreover, The Thai version of FACT-L closely resembled the original instrument and was widely used in several global clinical studies for Thai patients with lung cancer (backward translation).

This application was designed using an interactive pattern. For example, after staff received information from patients, they would manage their cases by replying instant advice or directly contact by phone depending on the severity of patient-reported outcome (PRO) results.

#### Cohort 2

##### i-health-related-QoL

The FACT-L was completed at baseline (before treatment), day 1 of the first follow-up (weeks 3–4), and third follow-up (3 months after randomization) of the treatment. QoL scores were reported by the domain, total FACT-L (sum of all five domains), total FACT-G (sum of PWB, EWB, SWB, and FWB), and the Trial Outcome Index (TOI; sum of PWB, FWB, and LCS). Higher scores represent better QoL. The quality of life data were reported as zero in case of those patients who were reported dead during the follow-up period for handling of missing data. Additionally, adverse events from the treatment were evaluated using the CTCAE, version 4.0. However, only the quality of QoL data from the validation of the Thai version of FACT-L was analyzed for the end point in this study. Furthermore, survival data were also obtained.

##### ii-tool-performance-analysis

The correlation between this tool (PRO scores) and clinical progression by imaging was analyzed using Fischer's exact test calculator for a 2 × 2 contingency table.

### Sample Size

**Figure 1:**
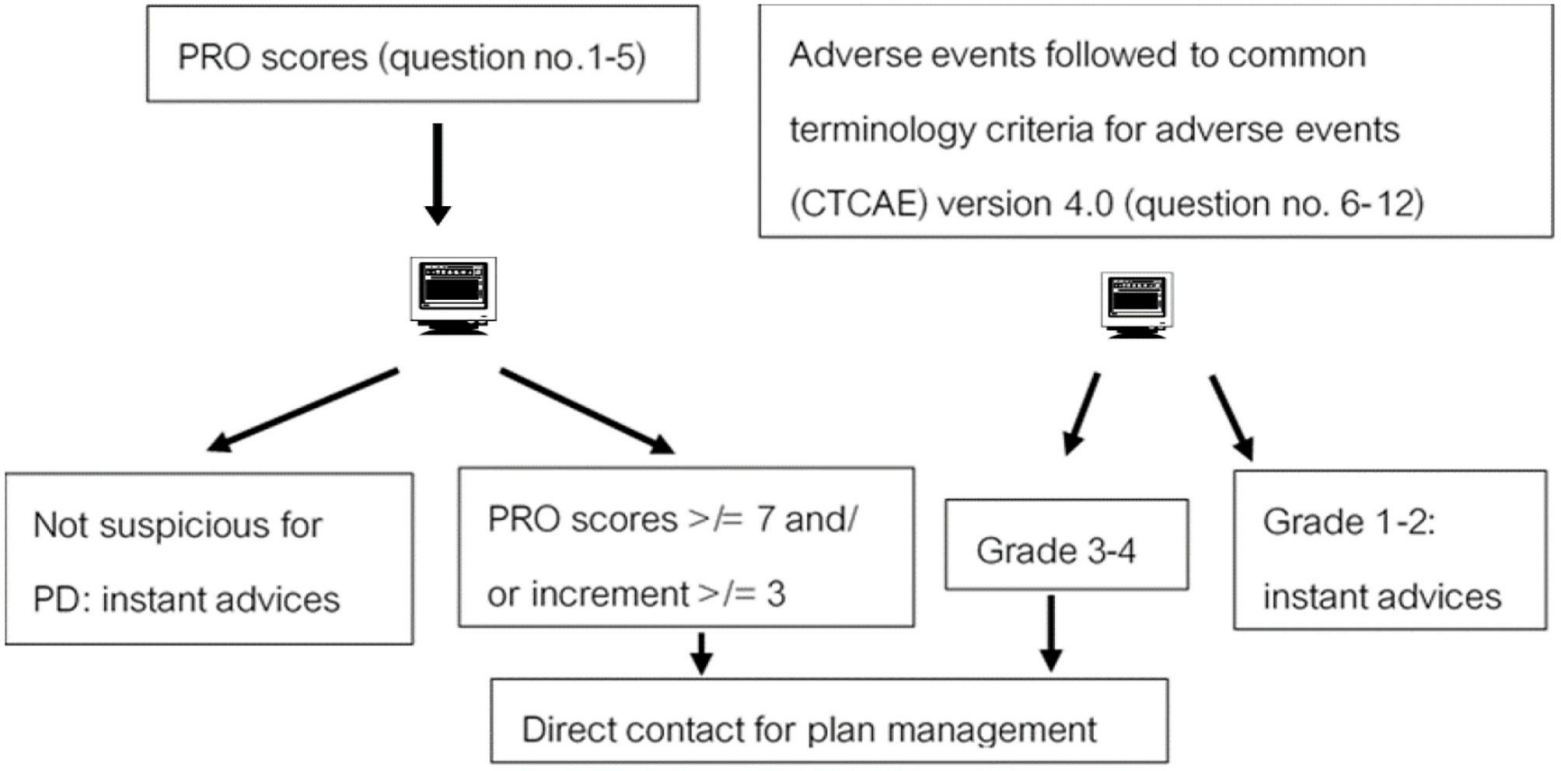
A flow chart of responding toward PRO results.

To test the performances of this mobile application-based PRO, integration in the mobile application for real-time patient outcome follow-up was investigated. From related studies Denis et al. (3, 4), the criterion of suspicious progressive disease (PD) by web-based PRO was defined by total scores of questions numbers 1–5 ≥ 7 and/or increment of scores ≥ 3 (10–12). Therefore, we calculated the sample size, which was at least 27 patients to identify the specificity of 0.93 as the original tool. Moreover, the performances were reported as sensitivity, specificity, positive predictive value (PPV), and negative predictive values (NPV) for PD. The chi-square test was used in 2 × 2 tables to assess the statistical association between the PD and the standard follow-up protocol. All tests were two-sided, and the *p*-value was considered significantly different when <0.05. A flow chart of responses toward PRO results is shown in Figure 1.

### Data Security

**Figure 2:**
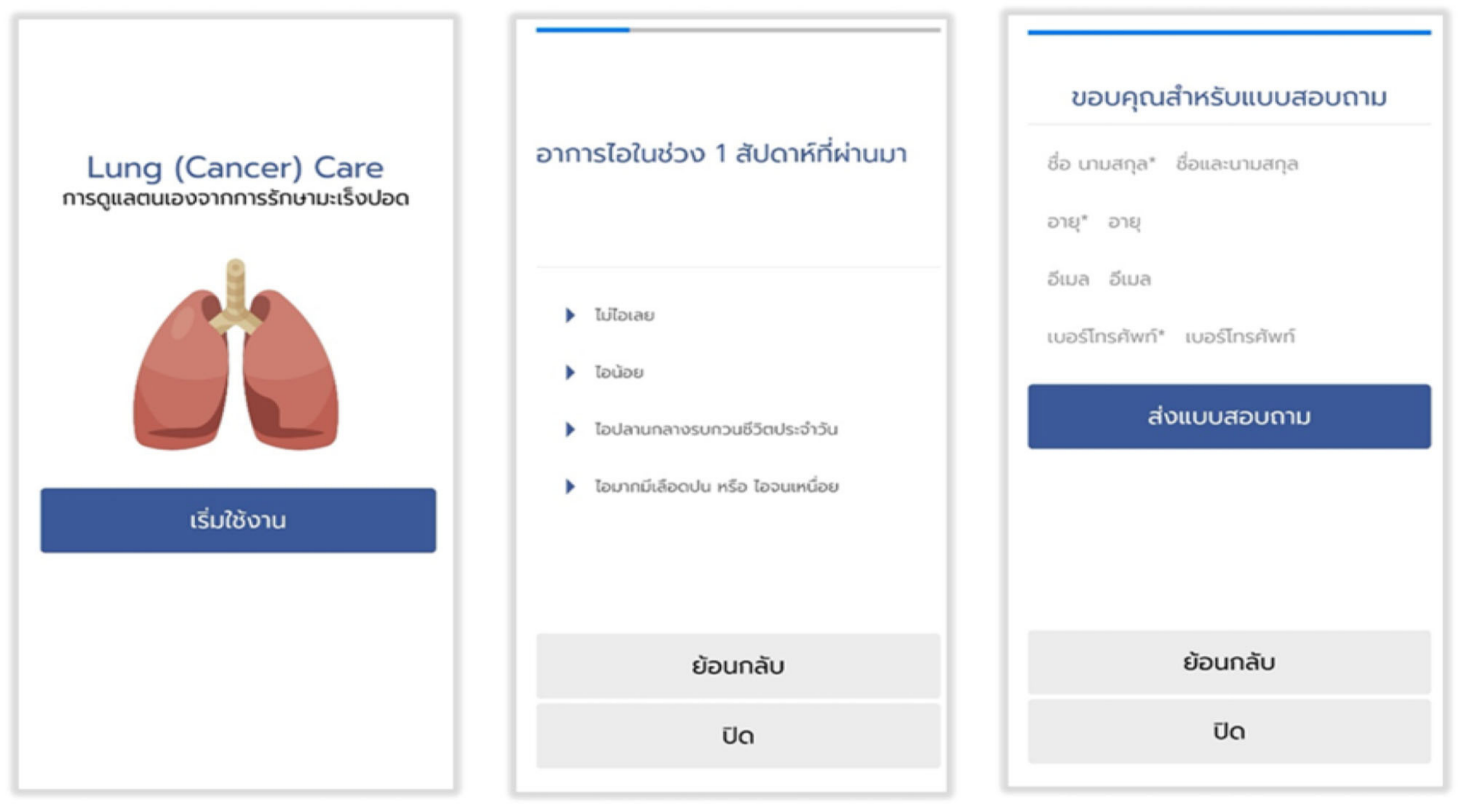
Interface features of lung cancer care application in Thai version.

The Lung Cancer Care application was only accessed by registered and authorized users. Each user was assigned a unique identifier and password. Study owners were able to review all of the data of enrolled patients in the study. All patient data held in Lung Cancer Care were de-identified or anonymized when entered into the database (Figure 2).

### Outcome Assessment and Statistical Analysis Methods

The clinical outcomes included QoL and OS, which are defined below.

- Quality of life was assessed at the OPD visit in both the groups at baseline (before treatment), day 1 of the first follow-up (weeks 3–4), and third follow-up (month 3) of the treatment using FACT-L (scoring 0–136; higher scores represent better QoL). The analysis of changes in mean FACT-L used Turkey-Kramer honestly significant difference (HSD) indicated a statistically significant difference when the *p*-value was <0.05. The QoL data were reported as zero in case of those patients who were reported dead during the follow-up period for handling of missing data.
- Overall survival was the time from randomization to death from any causes. This end point was analyzed using the Kaplan-Meier method and Cox proportional hazard model, which revealed statistical significance when the *p*-value was <0.05.
- The tool performances were reported as sensitivity, specificity, PPV, and NPV for PD by Fisher's exact test in 2 × 2 tables.
- SPSS, version 22.0 was used for statistical analysis.

## Results

### Cohort 1

**Figure 3:**
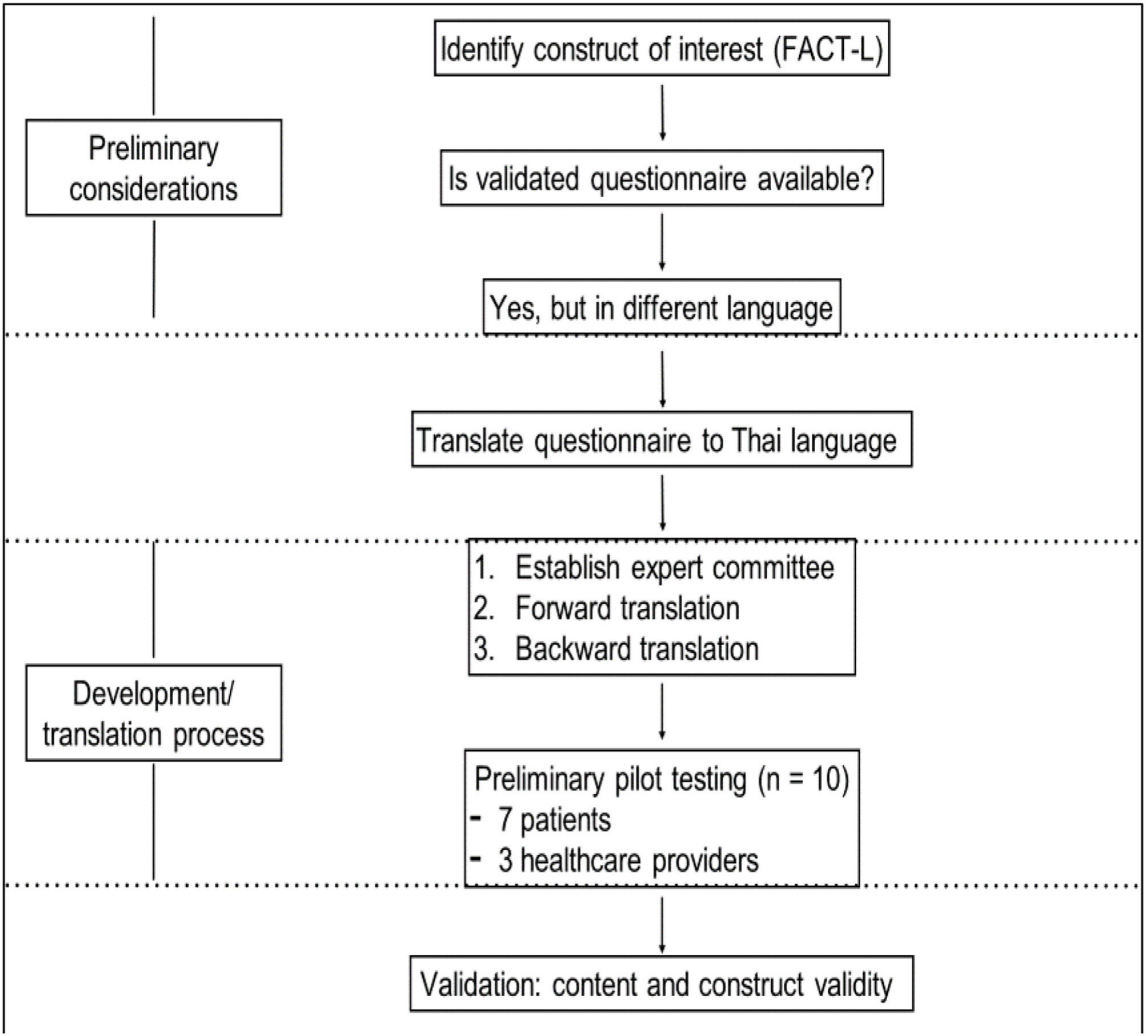
The tool development and translation processes.

Preliminary pilot testing for this smartphone application (Figure 3).

As with developing a new mobile application from April to June 2018, the prefinal version of the translated questionnaire was pilot tested on a small sample of the intended respondents (*n* = 10; 7 patients and 3 healthcare providers). After completion of the translated questionnaire, the respondent was asked verbally by an interviewer to elaborate what they thought on each questionnaire item and what their corresponding response meant. This process was repeated a few times to finalize the final version of the mobile application for using in valid across population.

### Cohort 2

#### HRQoL Score Between Two Groups

**Figure 4:**
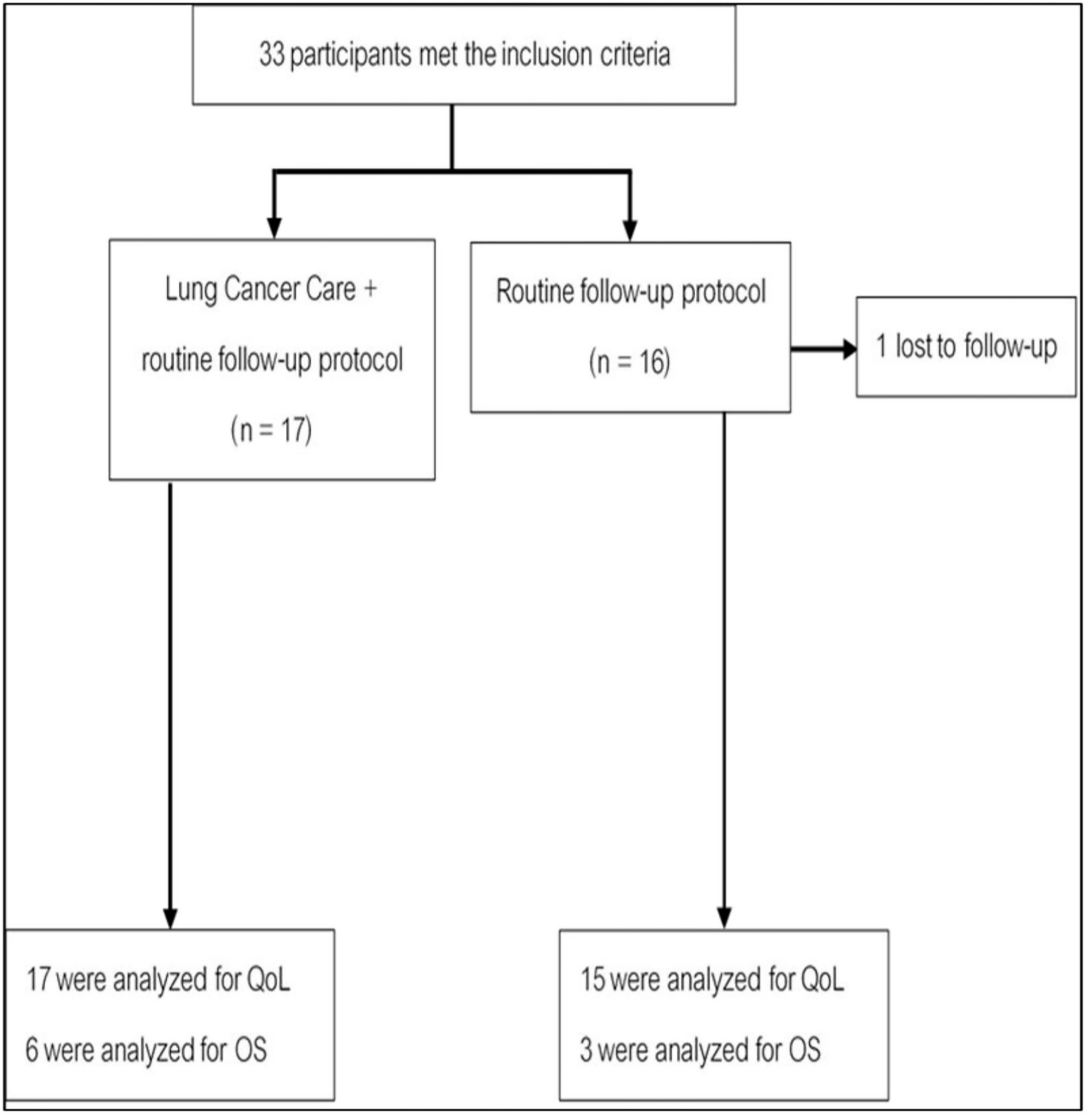
A consort diagram for enrolled patients in cohort 2.

Another 33 patients with advanced NSCLC, receiving first- or second-line systemic treatment were enrolled in cohort 2 of the study. In total, 17 patients were randomly assigned to use the Lung Cancer Care application (intervention arm), and 16 patients were monitored with routine follow-up care (control arm). One patient from the control arm was lost to follow-up (Figure 4). The baseline of patient's characteristics was well balanced between the two arms (Table 2). The median age was approximately 60 years old. Two-thirds of the patients were men. Most histology subtypes of enrolled patients presented adenocarcinoma.

**Table 2:** Baseline characteristics and Functional Assessment of Cancer Therapy-Lung (FACT-L) scores of both groups.

**Characteristic**	**Mobile application arm (*n* = 17)**	**Routine follow-up arm (*n* = 16)**	***p-*value**
**Sex, No. (%)**			
Male	9 (53)	12 (75)	
Female	8 (47)	4 (25)	
Median age (SD), years-old	61.6 (10.5)	63.8 (12.7)	
**Performance status, No. (%)**			0.99
0	1 (5.9)	1 (6.25)	
1	9 (52.9)	8 (50)	
2	4 (23.5)	4 (25)	
3	2 (11.8)	2 (12.5)	
4	1 (5.9)	1 (6.25)	
**Histology subtype, No. (%)**			0.29
Adenocarcinoma	17 (100)	15 (93.75)	
Small cell lung cancer	0	1 (6.25)	
**Technical skill, No. (%)**			0.86
Able	9 (53)	8 (50)	
Family aids	8 (47)	8 (50)	
**Active treatment, No. (%)**			0.32
Chemotherapy	9 (52.9)	12 (75)	
Targeted therapy	7 (41.2)	4 (25)	
Immunotherapy	1 (5.9)	0	
**Line of treatment, No. (%)**			0.92
1^st^ line	13 (76.5)	12 (75)	
2^nd^ line	4 (23.5)	4 (25)	
Symptom scores by application at baseline, mean (lower 95% mean, upper 95% mean, SE)	6.88 (4.78, 8.97, SE = 0.98)	7.25 (4.78, 9.38, SE = 1.01)	0.79
FACT-L at baseline, mean (lower 95% mean, upper 95% mean, SE)	90 (79.1, 101.1, SE = 5.4)	91.8 (80.4, 103.1, SE = 5.6)	0.82
FACT-L after 3 months (mean, lower 95% mean, upper 95% mean, SE)	99.96 (88.0, 111.85, SE = 5.74)	106.0 (93.63, 118.37, SE = 5.97)	0.07
Difference in FACT-L score between baseline and 3 months follow-up	8.18 ± 0.34 (missing = 7)	15.92 ± 0.37 (missing = 7)	0.05^*^

^*^ = means comparisons for all pairs using Tukey-Kramer HSD.

**Figure 5:**
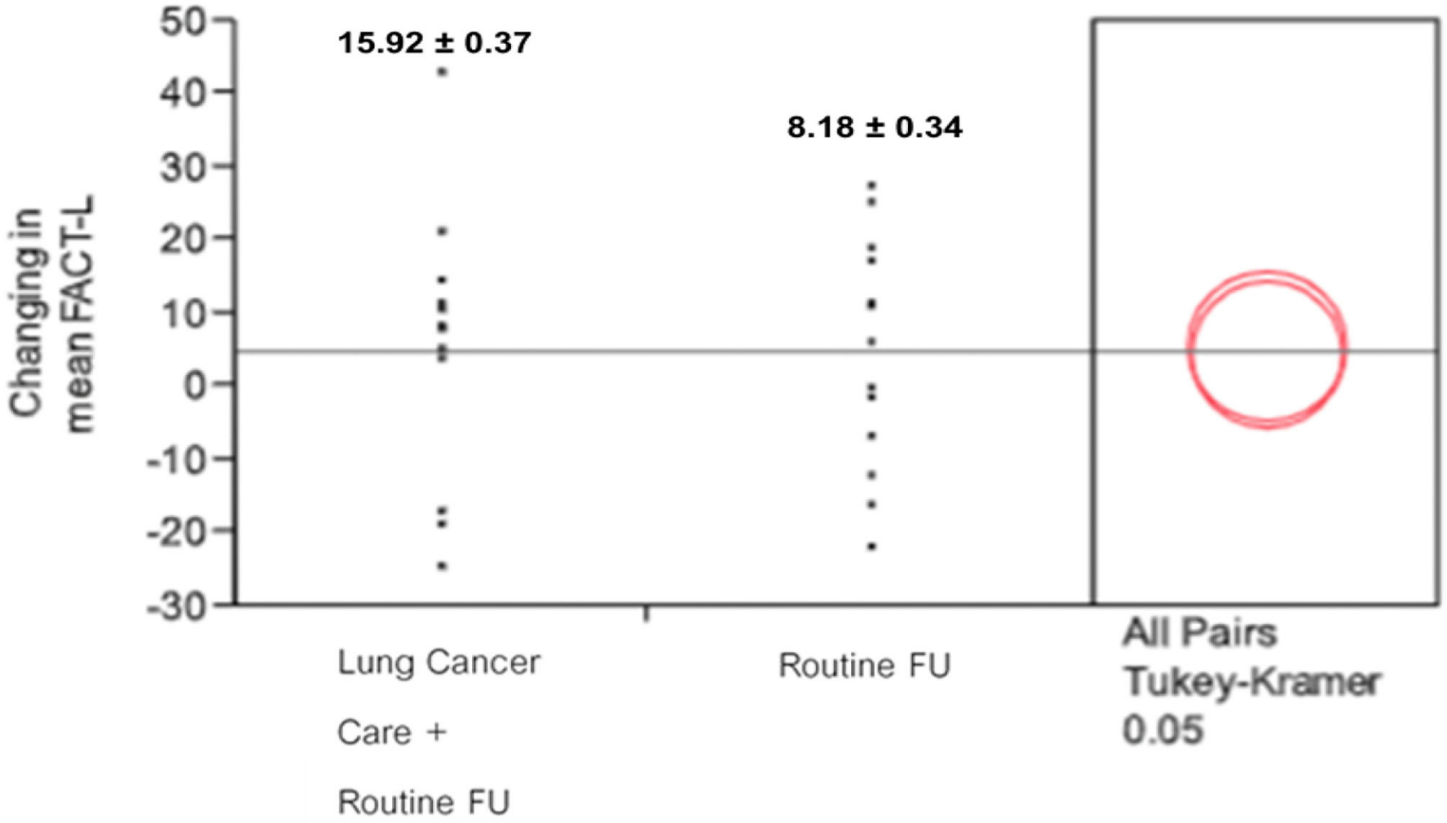
Changing in mean of FACT-L score between 2 arms.

In total, 32 patients (17 patients in the intervention arm and 15 patients in the control arm) were analyzed for QoL scores at baseline and 3 months after the randomization. The change in mean FACT-L scores at baseline and 3 months was compared between the two arms. The mean FACT-L score at baseline in the mobile application-based PRO arm and routine follow-up arm was similar (90.08 ± 5.66 vs. 91.78 ± 5.26*, p*-value = 0.82). Patients in the mobile application group had higher FACT-L scores at 3 months than patients with the routine follow-up arm (106 ± 5.97 vs. 99.96 ± 5.74, *p*-value = 0.07). The difference of mean comparisons for all pairs are shown by Turkey-Kramer HSD in Figure 5, exhibiting a *p*-value of 0.05 suggesting a trend for improving QoL when adding Lung Cancer Care application to routine follow-up protocol during the active cancer therapy among patients with advanced NSCLC.

### Overall Survival

**Figure 6:**
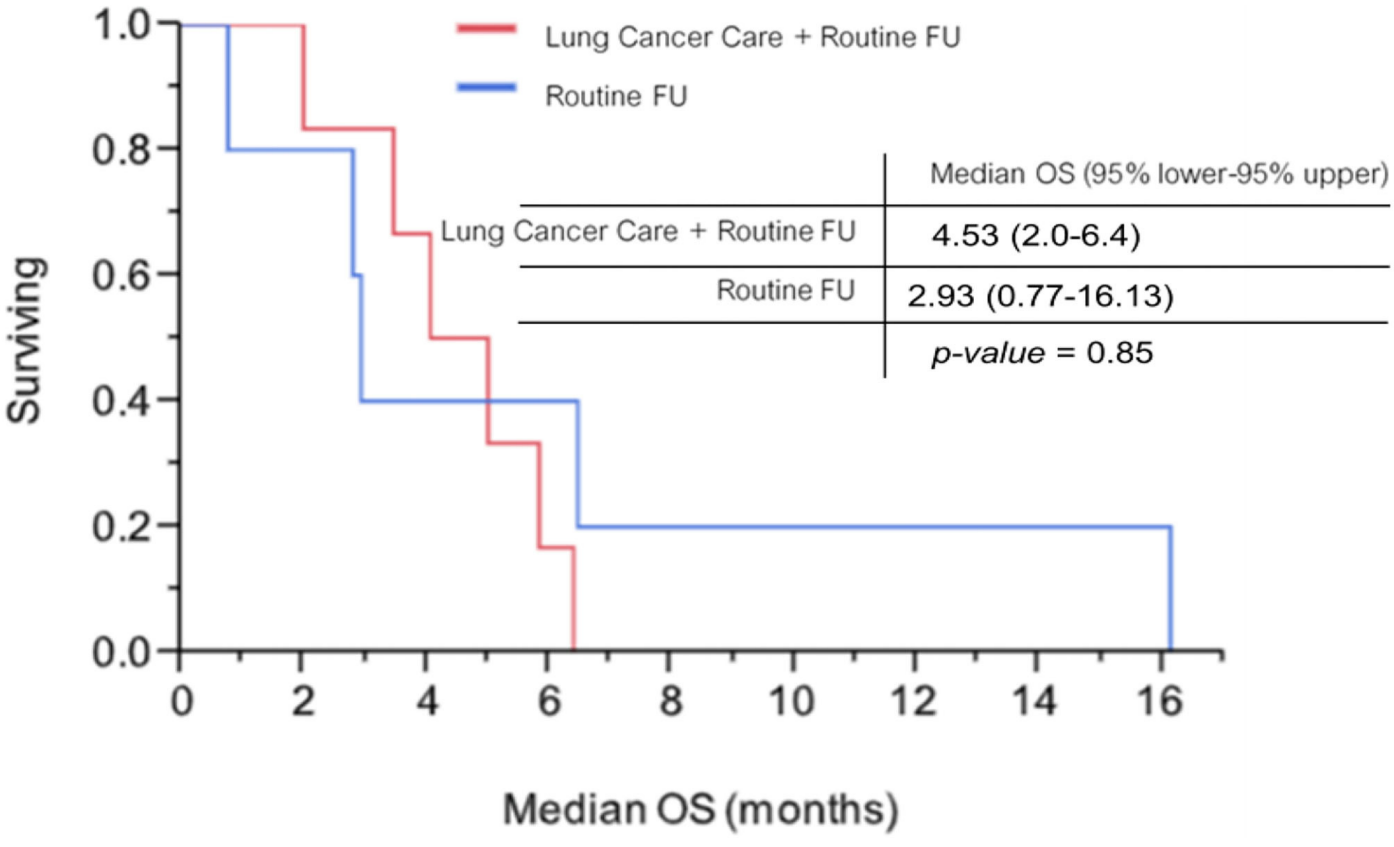
Overall survival compared between the two arms using Kaplan-Meier analysis.

On 31 March 2019, as the cutoff date, the median follow-up time was 5.43 months. Six of 17 patients with the mobile application arm and 3 of 15 patients with the routine follow-up arm were included for Kaplan-Meier analysis. Median OS among patients with the mobile application arm and patients with the routine follow-up arm were 4.53 months (95% lower−95% upper, 2.0–6.4 months) and 2.93 months (95% lower−95% upper, 0.77–16.13 months), respectively. No statistically significant difference was found between the two arms (*p*-value = 0.854; Figure 6).

### The Correlation Between Tool and Clinical Progression

At the time of the study, the cutoff date (31 March 2020), 10 of 17 patients from cohort 2 developed evidence of PD by imaging (CT chest with upper abdomen) or suspicion of PD by a tool.

The definition of PD by imaging (CT scan) was followed by Response Evaluation Criteria in Solid Tumors (RECIST), version 1.1. The criterion of suspicion of PD by a tool was referred from related studies (10, 11), for which the completed total QoL score (PRO scores) on the third visit was ≥7, or incremental score calculation was ≥3 (Figure 1).

The relationship between PD by imaging and PRO score is shown in Table 3. Two patients had true positive, 5 patients had true negative, 1 patient had false positive, and 2 patients had a false negative. The correlation between PRO scores and imaging for detecting PD was demonstrated by sensitivity, specificity, PPV, and NPV, namely, 50, 83.3, 66.7, and 70%, respectively. No statistically significant difference was found in the proportions of PD using tools and imaging (*p*-value = 0.5; Table 3).

**Table 3:** Relationship between PD by imaging and patient-reported outcome (PRO) score (tool performances analysis).

**Suspicion of PD by tool**	**PD by CT scan**		
	**Positive**	**Negative**	**Total**
Positive	2	1	3
Negative	2	5	7
Total	4	6	10
Fisher's Exact (2-tail)			0.5

## Discussion

This study is aimed to contribute evidence of using a smartphone application-based PRO to support patients with advanced NSCLC as they cope with the challenges of the disease and side effects from systemic treatments.

The Lung Cancer Care application was the first Thai version of the smartphone-based PRO. Our application developers built the Lung Cancer Care application with features and functionality regarding Health Insurance Portability and Accountability Act-compliant healthcare application development by using NetBeans 8.2. This application was designed to be simple and user-friendly displaying a clear front and large buttons for an android operating system (Supplementary Material). Interestingly, more than 50% of patients from our cohort had good technical skills using smartphones, which was similar to the US population. The evidence from the US National Cancer Institute in 2016 showed that 68% of Americans were able to use a smartphone by themselves.

For the validity of this tool, the questionnaires from validation of the Thai version of FACT-L were contained in this mobile application. In addition, the FACT-L questionnaire was initially translated from the original language to Thai (forward translation). Moreover, the Thai version of FACT-L closely resembles the original instrument and has widely been used in several global clinical studies for Thai patients with lung cancer (backward translation). Therefore, this mobile application was assumed to be validated. Furthermore, this tool was pilot tested using a small number of patients and healthcare providers. Feedback from healthcare providers and physicians using this mobile application confirmed that ease of familiarization with an application's user interface was of key importance for continued use. Currently, feedback has been positive with users finding the mobile application was easy to navigate and manipulate. However, regarding the small number of enrolled patients for pilot testing, a relatively large number of sampling errors may reduce the statistical power needed to validate this tool. Additionally, the larger number of enrolled patients in cohort 1 could have provided more information for developing the user-friendly interface design and building a robust platform with a commitment to innovation and ensuring the quality of the tool. Moreover, the reliability test of this tool should be further explored. Our study did not conduct the test-retest reliability because these questionnaires were constructed to measure transitory attributes, such as quality of recovery from systemic treatment. Therefore, the test-retest reliability might not be applicable as the changes in patients' responses between assessments were reflected in the instability of their patients. Additionally, the inter-rater reliability was not evaluated as this tool involved only a few raters completing the same instrument for each patient.

Our study demonstrated that patients using the Lung Cancer Care application had a trend for better QoL scores than patients who were monitored with routine care. It may be that this application was user-friendly, easily accessible, and indicated real-time to response. Therefore, this tool could narrow the existing communication gaps between healthcare providers and patients concerning their self-care and adverse events monitoring, for which it enhanced an efficacy of monitoring patients' valued outcomes, improving healthcare quality, and providing appropriate treatment options from patients' perspectives. However, the development of this application should be considered. The completion of the validation and transformation process of novel applications should be done initially.

Another study from China evaluated using a mobile application (e-support program) vs. routine care among 108 patients with breast cancer who underwent chemotherapy from two university-affiliated hospitals. This study was a 6-month, single-blinded, multi-center, randomized, and parallel-group superiority design. The results for comparing QoL scores between two arms have not been published yet. However, the strengths of this study were an innovative mobile application intervention, namely, Breast Cancer e-Support, with a rigorous study design and theoretical framework. The Breast Cancer e-Support program had four modules, such as a learning forum, a discussion forum, an ask-the-expert forum, and a personal stories forum (5).

Furthermore, two-thirds of patients in cohort 2 were selected for tool performance testing. Our study method for tool performance analysis was similar to related studies (10, 11) using the correlation between PRO scores and clinical progression from imaging. However, our tool exhibited lower performances than the original tool from related literature (Dr. Denis) (Table 4).

**Table 4:** Comparison of tool performances for detecting disease progression between Lung Cancer Care application and previous patient-reported outcome (PRO) international version.

**Parameter**	**Lung cancer care 2019**	**Fabrice Denis (3) 2014**
N	10	41
Sensitivity	50%	86%
Specificity	83.3%	93%
PPV	66.7%	86%
NPV	70%	93%

Our tool showed lower sensitivity, specificity, PPV, and NPV than related studies (10, 11) (Table 4). It may be that our study involved small samples, which an average was <5 patients on each cell as shown in Table 3. For these reasons, the Fischer's exact test was used for statistical analysis, which was a different statistical method from related studies. Moreover, a patient in our cohort developed brain metastases with mild hemiparesis, for which the neurological symptom was not included in the symptom scores. However, we concluded that our tool performance was invalid for predicting disease progression.

Regarding short-term follow-up, the difference in median OS could not be shown between the two arms. Moreover, median OS results from our study were too short when compared with other clinical studies and more than 50% of patients on each arm were censored from survival analysis.

We postulated that the negative results from our study were due to the following reasons: (1) small sample size, (2) short median time to follow-up, and (3) contamination from the patients with poor performance status in this trial (≈20%). This affected the tool to not properly discriminate among prognostic patients (Table 5).

**Table 5:** Demonstrated differences in patient characteristics between our study and related study (10).

**Parameter**	**Lung Cancer Care 2019**	**Fabrice Denis (10) 2017**
N	33 (planned 136)	121
Median follow-up	5.43 months	9 months
**PS**
0–2	81.8%	100%
>2	18.2%	-
Mean FACT-L at baseline (SD)	91.7 (8.5)	95.6 (16.7)

## Limitations

The Lung Cancer Care application study constituted only the pilot phase at Phramongkutklao Hospital. Regarding the small sample size in this study, the relatively small sampling errors may have increased the statistical power needed to validate this tool. We expect to increase the number of subjects so that this possibility could be investigated in a future large-scale prospective study.

Moreover, the dual challenges of large-scale distributed cloud-computing applications and massively multicore hardware platforms will be considered to develop a robust application and improve the accuracy of its evaluation results. Additionally, the integration of social functionality remains limited, such as real-time response. How to better facilitate communication among medical oncologists, internists, general practitioners, nurses, and patients should be studied in the near future. In addition, remote follow-up of patients *via* the line application is another feature that could be explored in the case of emergency treatment or when those patients are unable to attend the tertiary center for follow-up treatment.

## Conclusion

The Lung Cancer Care application constitutes the first smartphone-based PRO in Thai showing a trend toward improved quality of QoL among patients with advanced NSCLC. The knowledge gained from this study could lead to a better understanding of the role of self-efficacy and social support to develop a Lung Cancer Care application. The results from our study may help to further advance research regarding the use of electronic-based methods to improve QoL and psychological wellbeing for patients with lung cancer. Importantly, this tool is able to minimize the risks of exposure to COVID-19 in the hospital.

## Data Availability Statement

The original contributions presented in the study are included in the article/Supplementary Material, further inquiries can be directed to the corresponding author.

## Ethics Statement

The studies involving human participants were reviewed and approved by Institutional Review Boards, Royal Thai Army Medical Department Phramongkutklao Hospital. The patients/participants provided their written informed consent to participate in this study.

## Author Contributions

Material preparation, data collection, and analysis were performed by NP and KU. The first draft of the manuscript was written by NP and KU and all authors commented on previous versions of the manuscript. All authors contributed to the study's conception, design, read and, approved the final manuscript.

## Funding

This study received funding from the Department of Medicine, Phramongkutklao Hospital, Bangkok, Thailand.

## Conflict of Interest

The authors declare that the research was conducted in the absence of any commercial or financial relationships that could be construed as a potential conflict of interest.

## Publisher's Note

All claims expressed in this article are solely those of the authors and do not necessarily represent those of their affiliated organizations, or those of the publisher, the editors and the reviewers. Any product that may be evaluated in this article, or claim that may be made by its manufacturer, is not guaranteed or endorsed by the publisher.
