# Supplementary material for: A Pilot Study of Using Smartphone Application vs. Routine Follow-Up for Patient Care in Advanced Non-Small Cell Lung Cancer During the COVID-19 Pandemic Era
Source: Front Med Technol. 2022 Jun 21;4:900172. doi: 10.3389/fmedt.2022.900172 (PMC9253575; doi:10.3389/fmedt.2022.900172)

10.3389/fmedt.2022.900172

Supplementary Online Content

Electronic infrastructure

Our medical application developers built this application with features and functionality regarding HIPAA-compliant healthcare application development using NetBeans 8.2, which is a java integrated development environments (IDE) for writing the application on a mobile phone. The NetBeans platform provides a modular fashion, well developed windowing system, a service provider application programming interface and persistence mechanism, thereby promoting excellent programming practices.

A Lung Cancer Care application was designed to be simple, user-friendly with clear font, and large buttons for an android operating system. Enrolled patients could submit their information at any point, when desired. If any of the answers in the questionnaires met the severity thresholds previously determined, the patient would be able to contact their healthcare provider team promptly with a direct link by phone call or line application. A dashboard was created to display summaries of each patient's reports. The healthcare provider team was instructed to review and respond concerning their symptom reports within a few days depending on their severity, e.g., contacted patient, needed to go to the ER, no follow-up needed. Enrolled patients were monitored between visits via a smartphone application downloaded onto their personal device. The program contained in Lung Cancer Care application was managed to comply with data protection requirement and security standard (21 CFR part 11). All enrolled patient information was secured using encryption on the mobile device, in which android devices consisted of the Android 128-bit AES (GCM). All information stored on the smartphone application and transferred between systems was protected using secure transmission protocols and HIPAA-compliant databases. All communication between the application and other systems used HTTPS.

NetBeans IDE enabled configuring options for its many areas of functionality as well as customizing its workspace using option window. The option windows were enabled to set general IDE settings and edit any of the IDE's configurable settings. Moreover, inherent support was available for testing NetBeans platform application within the NetBeans platform's built-in harness scripts. Thus, functional and unit tests were supported out of the box. The simplification of testing infrastructure was referred to as "simpletests", which simplifies the setup of mandatory work.

Supplementary Online Content 1

Example of 11 PRO questions showing the real web-based interface

Question no.1: Fatigability

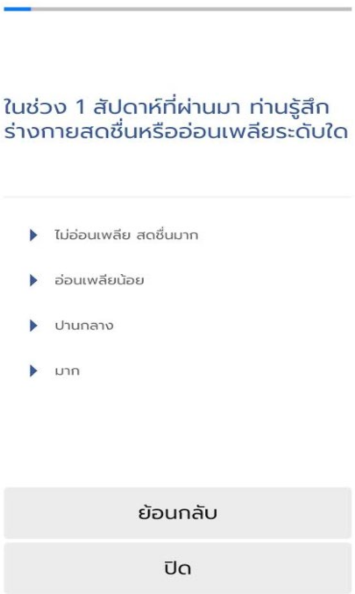

Question no.2: Appetite

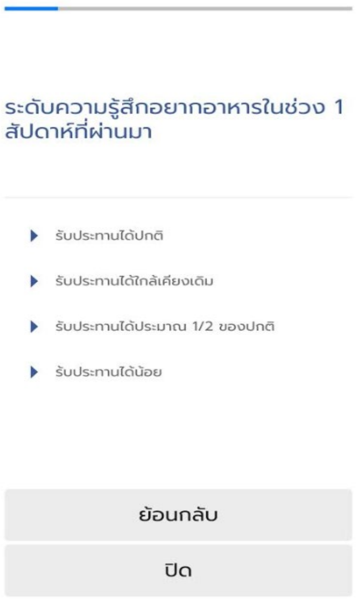

Question no.3: Cough

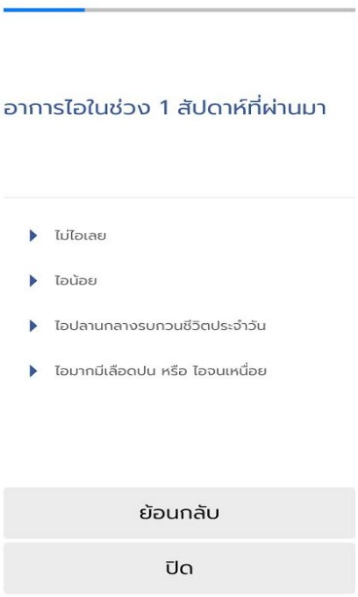

Question no.4: Dyspnea

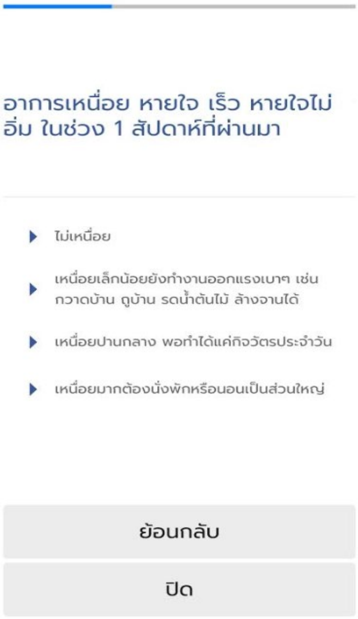

Question no.5: Pain

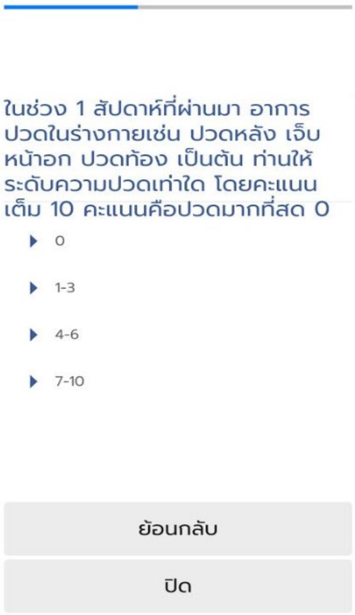

Question no.6: Diarrhea

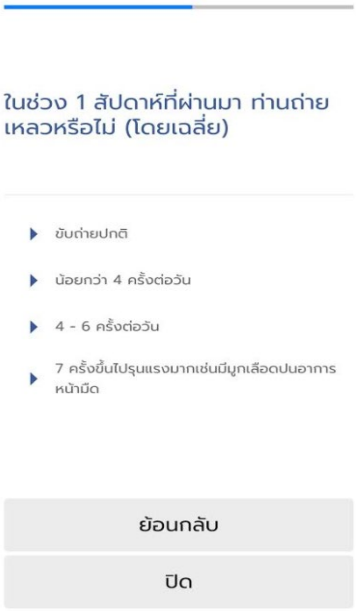

10.3389/fmedt.2022.900172

Question no.7: Rash, Skin lesions

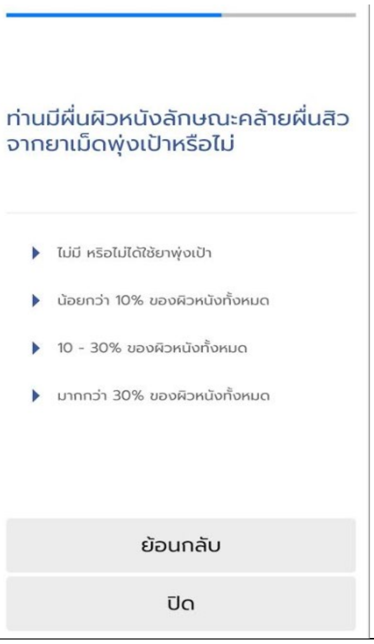

Question no.8: Paronychia

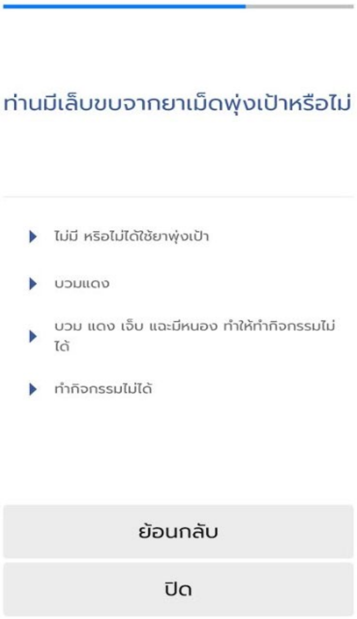

Question no.9: Mucositis/oral ulcer

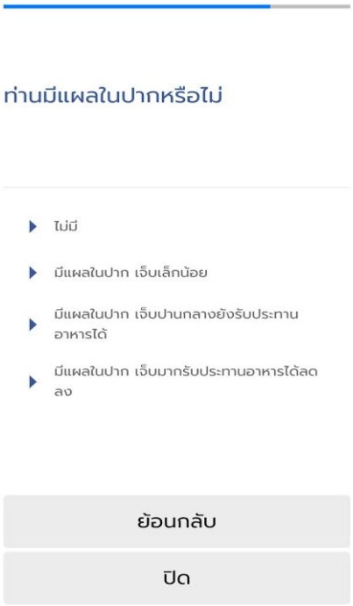

Question no.10: Numbness

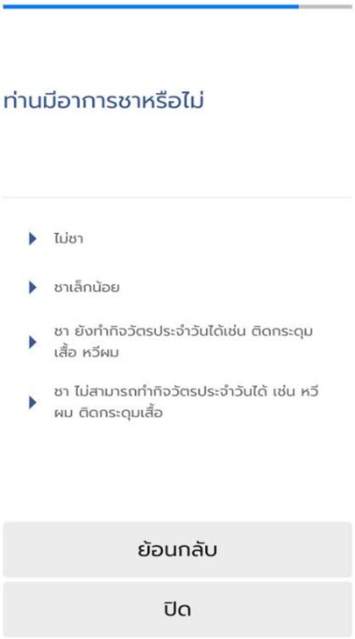

Question no.11: Fever

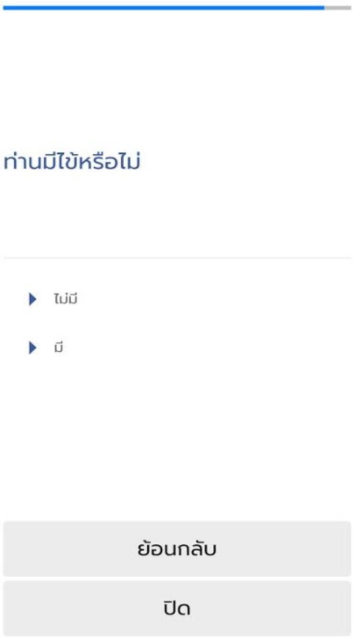

Supplement: Supplementary file 1 [file Data_Sheet_1.pdf]
